# Modelling control of *Schistosoma haematobium* infection: predictions of the long-term impact of mass drug administration in Africa

**DOI:** 10.1186/s13071-015-1144-3

**Published:** 2015-10-22

**Authors:** David Gurarie, Nara Yoon, Emily Li, Martial Ndeffo-Mbah, David Durham, Anna E. Phillips, H. Osvaldo Aurelio, Josefo Ferro, Alison P. Galvani, Charles H. King

**Affiliations:** Department of Mathematics, Case Western Reserve University, 10900 Euclid Avenue, Cleveland, Ohio USA; Center for Global Health and Diseases, School of Medicine, Case Western Reserve University, 10900 Euclid Avenue, Cleveland, Ohio USA; Department of Epidemiology, Yale School of Public Health, 60 College Street, New Haven, Connecticut USA; Schistosomiasis Control Initiative, Imperial College, Norfolk Place, St Mary’s Campus, London, UK; Universidade Catholica de Moçambique, Beira, Mozambique; Schistosomiasis Consortium for Operational Research and Evaluation, University of Georgia, Athens, Georgia USA

**Keywords:** Mathematical models, Theoretical/parasitology, Schistosomiasis/prevention and control, Drug therapy/organization and administration, Disease transmission, Infectious disease

## Abstract

**Background:**

Effective control of schistosomiasis remains a challenging problem for endemic areas of the world. Given knowledge of the biology of transmission and past experience with mass drug administration (MDA) programs, it is important to critically evaluate the likelihood that MDA programs will achieve substantial reductions in *Schistosoma* prevalence. In implementing the World Health Organization Roadmap for Neglected Tropical Diseases it would useful for policymaking to model projections of the status of *Schistosoma* control in MDA-treated areas in the next 5–10 years.

**Methods:**

Calibrated mathematical models were used to project the effects of different frequency and coverage of MDA for schistosomiasis haematobia control in present-day endemic communities, taking into account uncertainties of parasite biology and input data. The modeling approach in this analysis was the Stratified Worm Burden model developed in our earlier works, calibrated using data from longitudinal *S. haematobium* control trials in Kenya.

**Results:**

Model-based simulations of MDA control in typical low-risk and higher-risk communities indicated that infection prevalence can be substantially reduced within 10 years only when there is a high degree of community participation (>70 %) with at least annual MDA. Significant risk for re-emergence of infection remains if MDA is suspended.

**Conclusions:**

In a stable (stationary) ecosystem, *Schistosoma* reproduction and transmission are sufficiently robust that the process of human infection continues, even under pressure from aggressive MDA. MDA alone is unlikely to interrupt transmission, and once mass treatment is suspended, the prevalence of human infection is likely to rebound to pre-control levels over a period of 25–30 years. MDA success in achieving very low levels of infection prevalence is highly dependent on treatment coverage and frequency within the local human population, and requires that both adults and children be included in drug delivery coverage. Ultimately, supplemental snail control and significant improvements in sanitation will be required to achieve full control of schistosomiasis by elimination of ongoing *Schistosoma* transmission.

**Electronic supplementary material:**

The online version of this article (doi:10.1186/s13071-015-1144-3) contains supplementary material, which is available to authorized users.

## Background

Schistosomiasis is a chronic inflammatory parasitic disease caused by multi-year, infection by trematode blood flukes *Schistosoma* spp. These blood fluke parasites affect at least 240 million people worldwide [1]. Their control and possible elimination have been targeted recently by the World Health Organization in their 2020 Roadmap on Neglected Tropical Diseases (NTDs) [2], and by the 2012 London Declaration for Neglected Tropical Diseases (http://unitingtocombatntds.org/resource/london-declaration). However, effective control of schistosomiasis remains a very challenging problem for populations living in endemic areas of the tropical and sub-tropical regions of the world [3].

National and international schistosomiasis control programs are currently focused on expanding the use of mass drug administration (MDA) of the anti-schistosomal drug praziquantel to minimize infection-induced morbidity by reducing infection intensity among school-age children and high-risk adult populations [4]. This approach, termed preventive chemotherapy (PCT), has its limitations, in that parasite transmission can continue to occur, leaving populations at risk for reinfection and recurrent risk for disease [5–9]. The questions posed for the current modeling analysis are: Given what is known about the biology of parasite transmission, and given past experience with participation in MDA programs, how likely are we to achieve substantial reductions in *Schistosoma* prevalence, and over what time period? In particular, what will be the likely status of *Schistosoma* control in treated areas in the year 2020?

In the present study, we have used calibrated mathematical models to project the effects of different frequency and coverage of MDA for schistosomiasis control in present day endemic communities, taking into account uncertainties of parasite biology and input data (diagnostics). The modeling approach employed in this analysis is the Stratified Worm Burden (SWB) model developed in our earlier works [10, 11], and further refined in later reports [12]. The most recent version of our SWB model accounts for a number of ecological drivers that play key roles in *Schistosoma* transmission dynamics such as in-host parasite biology (worm mating, aggregation [13–15], and density dependent fecundity [16, 17]), human host population structure (demographics, spatial distribution [18–20]), and snail population dynamics. For the present study, the model was calibrated using an extensive data set (epidemiology, demographics, and snail environment) collected in control studies in *S. haematobium*- endemic communities of coastal Kenya [21–25]. In addition to worm distribution, the new SWB methodology simulates egg-release by different host worm burden strata. This plays an important role in estimating human-to-snail transmission (the force of infection (FOI) for snails). It is also important for predictive analysis of control programs in terms of projecting post-treatment prevalence outcomes, while accounting for the uncertainty of field diagnostics.

Our approach also allows the incorporation of model and data uncertainties into prediction uncertainty. Among these input uncertainties we include limited sensitivity and specificity of test diagnostics, the effect of stratified population sampling, parasite in-host biology (irregular egg-release [26]), and heterogeneities among human hosts in terms of parasite exposure, susceptibility to infection, and drug efficacy. Our modeling approach tracks two types of infection outcomes: (i) simulated egg-count test results (urine filtration for *S. haematobium*) that are typically utilized in MDA control program monitoring and evaluation, and (ii) the corresponding worm burden levels (expressed through dynamic SWB-variables) that can now be tracked with newer molecular diagnostics [27]. The resulting prevalence levels (“egg” vs. “worm”, *p*_*E*_ ≤ *p*_*W*_) derived from the model equations are used to evaluate the effectiveness of different control programs. As an illustration, we have compared our model projections to preliminary outcomes of the ongoing Schistosomiasis Consortium for Operational Research and Evaluation (SCORE) trials for gaining and sustaining control of *S. haematobium* in sub-Saharan countries (www.score.uga.edu, [10]).

## Methods

### 1. The SWB model

**Fig. 1:**
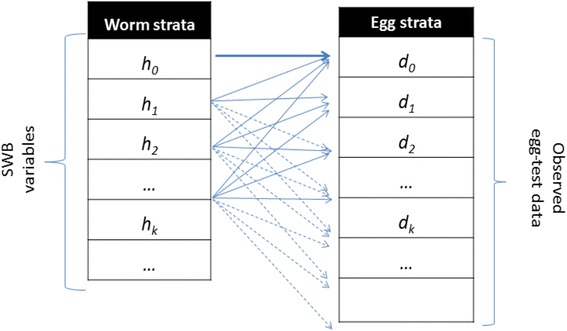
Schematic view of egg-release by SWB model population strata. Each host in *h*_*k*_ -stratum (*k* = 0, 1, 2, … ) carries, on average, *ϕ*_*k*_ mated worm couples, having fecundity factor *ρ*_*k*_. However, because egg-release/worm is random (in a negative binomial pattern with aggregation *r*_*k*_ = *r ϕ*_*k*_), test results for the *h*_*k*_ -stratum can actually be distributed over a broad range of egg-counts {*d*_*m*_}

We used a coupled human – snail SWB model with calibrated biological and transmission parameters to simulate long term impact of MDA control and evaluate its ability to reach a specific target prevalence or reduction of parasite burden. As detailed in our previous work [12], in the SWB model, a human population is divided into worm burden strata, *h*_*k*_(*t*), defined by a standard *worm burden increment, Δw*, with each stratum populated by human hosts carrying *k Δw* ≤ *w* < (*k* + 1) *Δw* adult worms (Fig. 1). Higher worm burden strata ({*h*_*k*_ : *k* ≥ 1} contribute to parasite transmission, while the lowest stratum (*h*_0_ ) does not (SWB details are further explained in Additional file 1, Additional file 2, Additional files 3, 4, and in Tables 1 and 2).

**Table 1:** System variables for the SWB model

SWB prevalence strata:	
*k Δw* ≤ *w* < (*k* + 1)*Δw*	{*h*_*k*_(*t*) : *k* = 0, 1, …}
with worm burden increment *Δw*	∑_*k*_ *h*_*k*_ = 1
Demographically structured SWB:	
Child	*h*_*k*_^*C*^(*t*)
SAC	*h*_*k*_^*S*^(*t*)
Adult	*h*_*k*_^*A*^(*t*)
Population densities per unit habitat:	
human	*H*(*t*)
SEI (susceptible, infected, patent) snail	*N*(*t*) = *x*(*t*) + *y*(*t*) + *z*(*t*)

**Table 2:** Demographic and biological parameters used for Stratified Worm Burden systems

Parameters	Name	Value
a. Demographic parameters for chldren (0–8 year), SAC (9–12 year) and adults (13+ year) for Mozambique		
Host turnover rates		
Child	*μ*_*C*_ = *τ*_*C*_ + *δ*_*C*_ (maturation + mortality)	0.13 + 0.01/year [34]
SAC	*μ*_*S*_ = *τ*_*S*_ + *δ*_*S*_ (maturation + mortality)	0.33 + 0.02/year [34]
Adult	*μ*_*A*_ (mortality)	0.026/year [34]
Demographic sources		
Child	*S* ^*C*^ = {*b*_*C*_, 0, 0, …}; per capita birth rate	*b*_*C*_ = 0.04/year [34]
SAC	*S* ^*S*^ = *τ*_*C*_{*h*_0_^*C*^, *h*_1_^*C*^, …}	
Adult	*S* ^*A*^ = *τ*_*S*_{*h*_0_^*S*^, *h*_1_^*S*^, …}	
Mean daily urine release		
Child		*U*_*C*_ = 700 mL^a^
SAC		*U*_*S*_ = 1100 mL^a^
Adult		*U*_*A*_ = 1300 mL [43]
b. Demographic parameters for chldren (0–20 year) and adults (20+ year) in Kenya		
Host turnover rates		
Child	*μ*_*C*_ = *τ* + *δ*_*C*_ (maturation + mortality)	0.05 + 0.003/year [34]
Adult	*μ*_*A*_ (mortality)	0.02 − 0.03/year [34]
Demographic sources		
Child	*S* ^*C*^ = {*b*_*C*_, 0, 0, …}; per capita birth rate	*b*_*C*_ = 0.032/year [34]
Adult	*S* ^*A*^ = *τ*{*h*_0_^*C*^, *h*_1_^*C*^, …}	
Mean daily urine release		
Child		*U*_*C*_ = 1100 mL^a^
Adult		*U*_*A*_ = 1300 mL [43]
c. Snail parameters		
Snail mortality	*ν*_*S*_	2.6/year^b^
Worm mortality	*γ*	0.2/year [44]
Recovery/conversion rate	*r*	¼ weeks^b^
Patency conversion fraction	*c*	0.05–0.2^c^

^a^from http://www.thepostnatal.com/2011/06/urine-output-at-different-ages/

^b^from [23, 25] and Kariuki et al., unpublished data

^c^based on results of snail data calibration

**Fig. 2:**
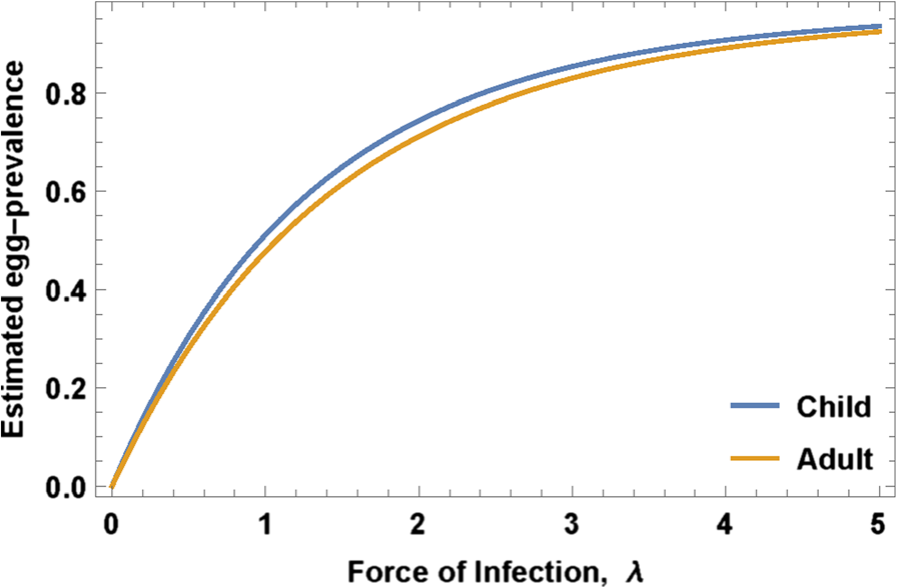
Egg-prevalence as function of human force of infection (FOI). Projected egg-prevalence curves *p*_*E*_(*λ*) are shown for typical median values of child and adult parameters (*ρ*_0_, *k*_0_, *r*) obtained via our calibration approach

Worms release eggs in an irregular random fashion, making precise diagnosis of worm burden difficult. Following [26], in our estimates of egg outputs, we assume a negative binomial (NB) distribution for egg-release by mated worms with density-dependent mean worm fecundity,

1 $$ {\rho}_k={\rho}_0\;{e}^{-k/{k}_0},\ \mathrm{f}\mathrm{o}\mathrm{r}\ k-\mathrm{t}\mathrm{h}\ \mathrm{stratum},\kern0.15em {h}_k $$

where *ρ*_0_ - maximal egg production/worm, *k*_0_ - crowding threshold, linked with an NB aggregation parameter *r*. The model parameters that require calibration are related to parasite biology (in-host worm fecundity factors {*ρ*_0_, *k*_0_, *r*}) and to snail-to human transmission (human FOI, *λ*) for different human population fractions (children and adults) having higher or lower risk of new infection (Fig. 2).

Human communities are coupled to snail transmission environment via two forces of infection: snail-to-human *λ* = *A z* (proportional to shedding snail prevalence *z*) and human-to-snail *Λ* = *Λ*(*E*) - a nonlinear function of combined human infectivity *E. E* is determined by a worm mating factor *ϕ*_*k*_ which is equal to the product of the estimated number of mated worm couples in the k-th stratum, (see equation (14) of Additional file 2), and worm fecundity (Eq. (1)), namely

2 $$ E={\displaystyle {\sum}_{k=1}^n{\rho}_k}{\phi}_k\;{h}_k={\displaystyle {\sum}_{k=1}^n{E}_k\;{h}_k} $$

Variable *E* represents the expected “egg release per host” for a given SWB community. Unlike random diagnostic tests, environmental egg-release *E* is viewed as deterministic process accumulating the random contributions of multiple human hosts within each community.

### 2. Egg-test and worm burden diagnostics for SWB

Egg count and hematuria diagnostics prove less reliable as population prevalence of infection decreases and average intensity of infection declines [28–31]. Therefore, to model the process of *infection*, we must account for the uncertainties of these standard diagnostics (as used in most present-day control programs) in assessing true treatment impact. Two types of diagnostic measures were simulated in our analysis: *p*_*E*_ - infection prevalence based on egg-tests (real or simulated) and *p*_*W*_ - positive (detectable) worm burden prevalence (as could be determined by highly sensitive circulating antigen tests [27]).

Both types of outcome are derived using a SWB formulation including a worm fecundity function (1) and aggregated NB egg-production by mated worms in order to account for egg-worm variations. The worm prevalence can be expressed through SWB variables {*h*_*k*_(*t*)} as

3 $$ {p}_W(t)=1-{h}_0(t) $$

Such a definition assumes that only infective strata (*h*_*k* ≥ 1_) are detectable by the molecular test (we recall that *h*_0_ is technically not an “infection-free” stratum but rather a “non-infective” one that is free of mated couples). If it happens that a particular molecular test has higher sensitivity (*e.g.*, one or 2 worms can be detected), definition (3) could be adjusted accordingly, e.g. *p*_*W*_(*t*) = 1 − *α h*_0_(*t*), where 0 < *α* ≤ 1 is the detectable fraction of *Δw* = 10 worms.

A key link between our model and projected MDA program egg-count outcomes is the simulated egg-test results whose values (egg-count distribution) depend on (i) the screened population sample drawn from the total population of interest (whether community or a population subgroup, typically these are sentinel school age children), (ii) the infectious status of the group/community given by its SWB distribution {*h*_*k*_(*λ*)}, (iii) the estimated egg-release per mated worm (or host) in different strata, as determined by fecundity function *ρ*_*k*_ of (1). There are two random steps in this procedure (i) random population sampling for screening and (ii) random egg-release by mated worms. The latter is assumed to be NB with mean = *ρ*_*k*_ (fecundity), and aggregation parameter *r* (details are provided in Additional file 2).

We make iterative use of the simulated egg-test results in the Bayesian Monte Carlo calibration procedure adopted here (see Additional file 3). They are also used to estimate the expected infection prevalence and intensity in simulated MDA control studies, specifically for a given SWB population {*h*_*k*_(*t*)} with fecundity *ρ*_*k*_ (1), mating factor *ϕ*_*k*_ (equation (14) of Additional file 2), and aggregation parameter *r*. The expected (mean) egg-test prevalence is given by

4 $$ {p}_E(t)=1-{\displaystyle \sum_{k=0}^n{\left(\frac{r}{r+{\rho}_k}\right)}^{\phi_k\kern0.1em r}{h}_k(t)} $$

(see [12] and Additional files 1 and 2).

For mixed SWB population systems with demographic fractions *H*_1_ + *H*_2_ + … = 1 (*e.g.*, child-adult, high-low risk), diagnostic prevalences *p* = *p*_*E*_ or *p*_*W*_ are given by

5 $$ p={H}_1{p}_1+{H}_2{p}_2+\dots $$

where *p*_*i*_ is the prevalence (3) or (4) of the *i*-th group.

### 3. Model calibration and Data Sources

The coupled (human-snail) SWB model employed in our current projections was calibrated using a detailed Kenyan dataset [8, 21, 23, 24, 32] which covers a broad range of host demographics, incidence, prevalence, and water use, as well as information about local snail abundance and geographic distribution. We employed a Bayesian calibration methodology that aims to estimate *likelihood weights* for different parameter choices by measuring the proximity of simulated egg-test to the real test data for a given community (see Additional file 3). The former (simulated test) depends on model parameters (*λ*, *ρ*_0_, *k*_0_, *r*), and we assign each choice of a combined parameter set selection its specific likelihood weight. The outcome of such calibration is creation of a posterior ensemble (distribution) of the most likely parameter values, specific for each community.

**Fig. 3:**
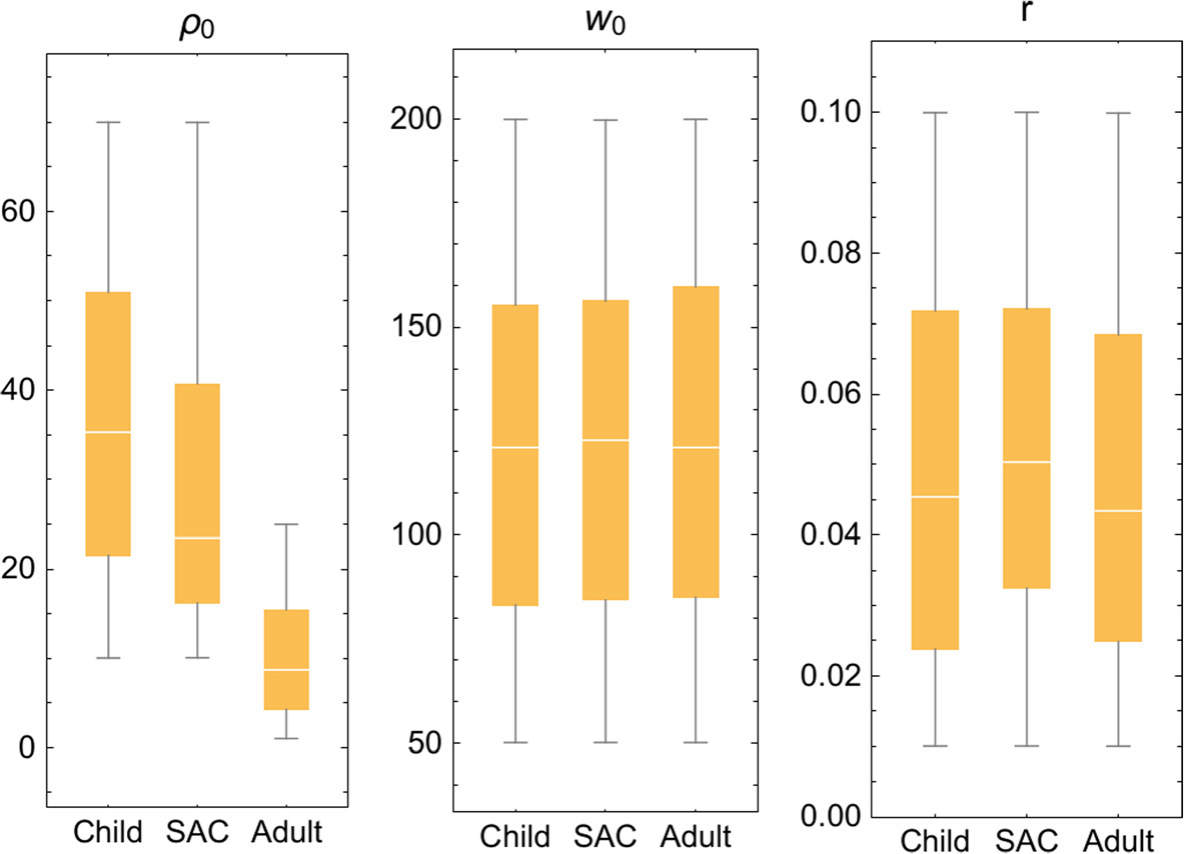
Distributed parasite biological parameters. Model parameters (*ρ*_0_, *k*_0_, *r*) estimated for 3 demographic groups (younger children, (child, 0-8 years old); mid-school age children (SAC, 9–12 years old), and adults (13+ years old) based on calibration using field data collected in the Msambweni sub-county area of coastal Kenya [32]. The individual parameters modeled are labeled at the top of each panel

While the ten Kenyan villages differed in terms of infection intensity and prevalence, we found that age-specific biological parameters maintained stable values regardless of location and transmission intensity. Building on this observation, we have ventured to apply Kenyan biological parameters (for selected demographic groups) to recent data from MDA-treated Mozambique populations, adjusting for local starting prevalence. We hypothesized that these biological features were a roughly constant feature of the parasite species, and could be considered comparable across endemic locations. Specifically, we chose to estimate our model’s biological parameters for 3 demographic groups (constituted from Kenyan data) that were consistent with the SCORE project’s age-group monitoring system in its operational research trials (younger children (0-8 yr), school age children (SAC, 9–12 yr sentinel age group), and adults (13+ yr)) and derived a posterior ensemble of biological parameters (*ρ*_0_, *k*_0_, *r*) for each monitored age group. The calibration results for these Kenya data are shown in Fig. 3 and Table 3. As expected, maximal fecundity, *ρ*_0_, decreased with human host’s age [33], while worm crowding threshold, *k*_0_, and aggregation, *r,* estimates stayed nearly constant across different human age groups. In calibrating our model system, we found remarkably consistent values for the three specific biological parameters, per age group, across the spectrum of low-risk to high-risk Kenyan villages. Because data from the Mozambique sites were more sparse in terms of individual age level infection and risk for reinfection, we have used these calibrated Kenyan parameter values for the simulation of Mozambique community outcomes.

**Table 3:** Calibrated *Schistosoma* biological parameters estimated for three demographic groups using the Kenyan dataset

	Child			SAC			Adult		
	ρ_0_	w_0_	k	ρ_0_	w_0_	k	ρ_0_	w_0_	k
Mean	37	120	0.048	30	120	0.052	10	120	0.047
SD	17	43	0.027	17	42	0.024	6.8	43	0.026

The next calibration step for coupled human-snail systems involves transmission inputs: FOI *λ* and coefficients *A* (snail-to-human) and *B* (human-to-snail). To estimate equilibrium FOI *λ* for a particular choice of biological parameters (*ρ*_0_, *k*_0_, *r*), we use Eq. (4) for a stationary SWB distribution {*h*_k_*(*λ*)}

6 $$ {p}_E=1-\sum_{k=0}^n{\left(\frac{r}{r+{\rho}_k}\right)}^{\phi_k\kern0.1em r}{h_k}^{\ast}\left(\lambda \right)=F\left(\lambda; {\rho}_0,{k}_0,r\right) $$

The right-hand side of Eq. (6) is a function of *λ* and the biological triplet (*ρ*_0_, *k*_0_, *r*), as illustrated in Fig. 2 for typical values of child/adult parameters (*ρ*_0_, *k*_0_, *r*). Solving this equation for a given observed prevalence, *p*_*E*_, we get equilibrium FOI, *λ*, and then transmission coefficients *A* and *B* can be estimated from the available human/snail demographic and infection data (see Additional file 3). In our analysis, we did not track change in transmission potential or FOI during MDA. In the Kenya study experience, even though the community egg output decreased, snail numbers and snail infection levels remained about the same despite good coverage of SAC and treatment of most high intensity infections [25]. There are non-linear aspects of contamination and miracidia-snail exposure [18], such that snail infection can persist at a significant rate even in the face of MDA. In most cases, we believe, significant change in *A* and *B* require environmental changes beyond the impact of MDA, and the impact of such interventions will be explored in future papers.

Some uncertainties are built in the system’s setup (*e.g*., multiple demographic/risk groups, transmission environment, diagnostics); others result from the Bayesian calibration procedure. Rather than “best-fit” parameters, we look for likely parameter choices. Thus, each community or population group is described by its “posterior” distribution in the parameter space. To simulate any particular outcome (*e.g.*, MDA control intervention) for a given community, we randomly sample its posterior distribution to generate an ensemble of “likely virtual communities” (parameter choices).

Then we compute the corresponding ensemble of worm burden and prevalence outcomes and assign each one its likelihood weight. The final result takes the form of a distribution of outcome values, and we report its statistics (mean, variance, confidence levels, etc.).

### 4. MDA control within SWB systems

The effect of drug treatment on an SWB population is to shift the treated fraction of stratum *h*_*n*_(*t*) to a lower-level stratum *h*_*m*_(*t*), where *m* ≈ *ε n*. Parameter *ε* is drug efficacy measured as fraction of adult worms surviving treatment [12]. For example, all strata in the lowest worm-range {*h*_*m*_ : 0 ≤ *m* < 1/*ε*} shift to *h*_0_ (*i.e.,* an effective clearing of patent worm infection). The next range {*h*_*m*_ : 1/*ε* ≤ *m* < 2/*ε*} goes into *h*_1_, etc. In numeric code, each MDA step is simulated as an “instantaneous event” due to the short duration of drug action (days) relative to slow time scale of transmission dynamics (months to years).

Computationally, dynamic SWB variables {*h*_*k*_ = *h*_*k*_(*t*_0_)} at the treatment time *t*_0_ are reinitialized to new (post-treatment) values {*h*_*k*_′} depending on the two MDA inputs: treatment coverage fraction (0 < *f* < 1) and drug efficacy *ε*. When the coverage fraction is relatively high (*f* ≈ 1 ), each stratum has approximately *f* treated plus (*1-f*) untreated hosts, so

7 $$ \begin{array}{l}{h_0}^{\prime }=\left(1-f\right){h}_0+f{\displaystyle \sum_{0\le m<1/\varepsilon }{h}_m}\\ {}{h_1}^{\prime }=\left(1-f\right){h}_1+f{\displaystyle \sum_{1/\varepsilon \le m<2/\varepsilon }{h}_m}\\ {}{h_2}^{\prime }=\left(1-f\right){h}_2+f{\displaystyle \sum_{2/\varepsilon \le m<3/\varepsilon }{h}_m}\\ {}\dots \end{array} $$

More generally, for a given coverage level 0 < *f* < 1, we draw a random sample of size *T* = *f H* = *T*_0_ + *T*_1_ + … + *T*_*n*_ from the total SWB human population *H* = *H*_0_ + *H*_1_ + … + *H*_*n*_ via a multinomial distribution with SWB probabilities *h*_*i*_ = *H*_*i*_/*H*. Then, we get estimated coverage fractions for each stratum {*f*_*i*_ = *T*_*i*_/*H*_*i*_} and the relationships among the Eq. (7) take the form

8 $$ {h_i}^{\prime }=\left(1-{f}_i\right){h}_i+{\displaystyle \sum_{i/\varepsilon \le m<\left(i+1\right)/\varepsilon }{f}_m{h}_m} $$

**Fig. 4:**
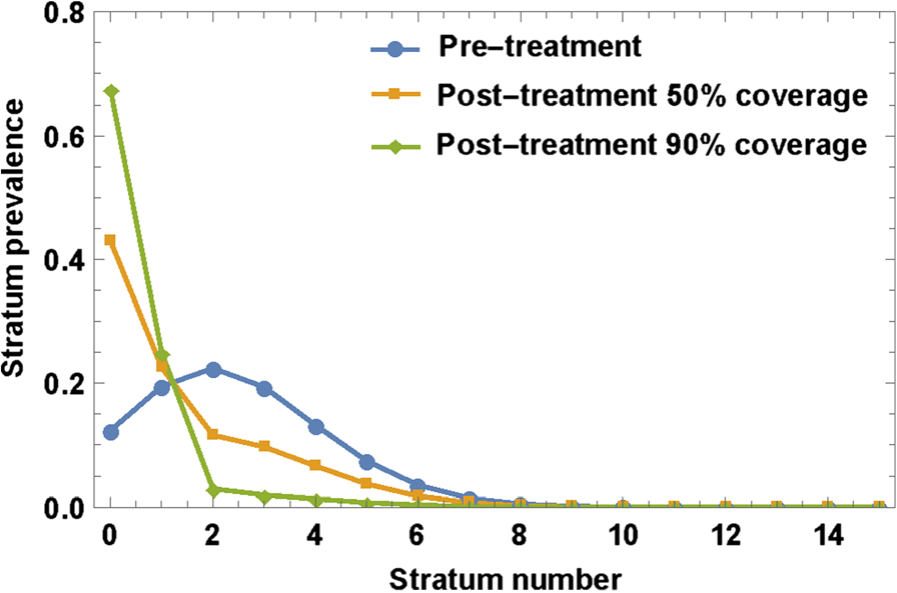
MDA effect on SWB strata. An MDA applied to SWB population would rearrange variables {*h*_*k*_} by shifting higher-burden stratum *h*_*k*_ → *h*_*ε k*_, depending on drug efficacy *ε* (worm survival rate after treatment), and treatment coverage fraction, *f*. The plot shows the predicted effect of different coverage fractions (*f = 50 %, 90 %)* on a typical SWB distribution, assuming *ε* = 0.75

Reinitialized system (7) or (8) is then solved over the following time-span until the next MDA “event”. The process runs according to a prescribed MDA control strategy in terms of frequency and age groups that have been targeted. Figure 4 illustrates typical MDA-mediated rearrangements of SWB stratum frequencies in a human community when given MDA at different treatment coverage levels (50 % vs. 90 %).

At each MDA step (or any other time *t*_*S*_), we can “diagnose” the state of our system (infection intensity, prevalence, etc*.*) by evaluating variables {*h*_*k*_(*t*_*S*_)} and using prevalence Eqs. (3)-(5). For long-term control predictions, we also take into account projected demographic changes of the local human populations [34].

### 5. Control strategies

**Fig. 5:**
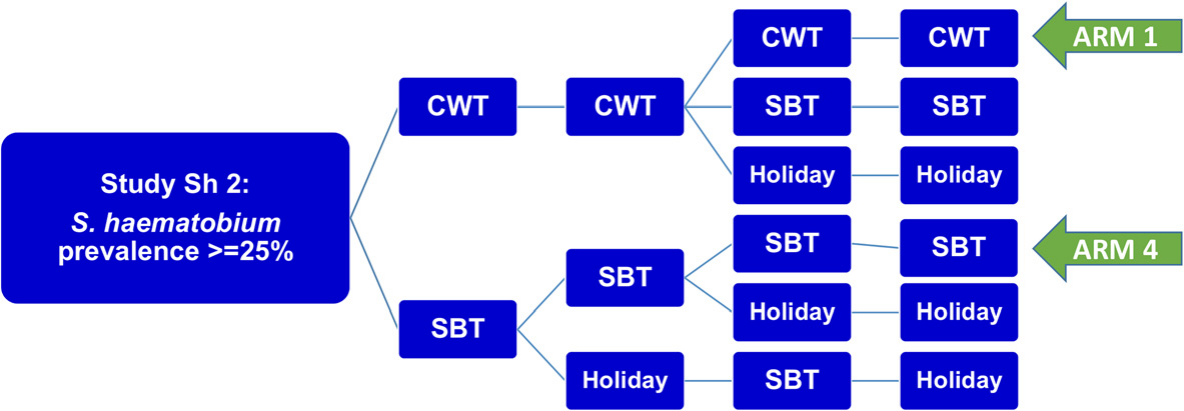
SCORE control strategies trial for high risk villages. The ongoing treatment strategies trial for high *Schistosoma* prevalence villages in SCORE project, comparing different frequencies of CWT, SBT, and treatment holiday intervals. The analysis in this paper focuses on the most aggressive arms (Arm 1 and Arm 4) of yearly high-risk village community-wide treatment (CWT) vs. school-based treatment (SBT)

We studied projections for two types of MDA control results: (i) short-term outcomes along the lines of the 5-year SCORE projects (see Fig. 5 for the SCORE ‘gaining control’ study design) and (ii) long term (30-year) outcomes of programs targeting extensive reduction of prevalence.

In each case, a virtual host community (village) is constructed from several human population age subgroups. The groups are represented by SWB systems and linked through birth-maturation source terms (see Additional file 1). The entire host community is coupled to a hypothetical snail habitat via calibrated transmission coefficients *A* and *B* that would maintain the locally-determined baseline (equilibrium) human-snail transmission pattern for infection.

For simulations of SCORE Project outcomes, we modeled outcomes for the three target age groups being monitored by the project: children 0–8 years old, SAC sentinel 9–12 year olds, and adults (13+), testing over a range of coverage levels observed in the field. Combining typical sub-Saharan rural demographics [34] with calibrated biological parameters, we generated an ensemble of virtual villages whose baseline infection and coverage levels were compatible with recent *S. haematobium* control data from Mozambique.

For longer term simulations, the modeled programmatic control target was set at using MDA to reach a ≤ 2 % infection prevalence. This level of prevalence was selected to approximate the successful long-term outcomes reported by large-scale national MDA-based schistosomiasis control programs of Egypt [35], China [36], and Brazil [37]. We monitored projected values for two types of diagnostics in our study: (i) simulated egg-test prevalence or its expected (mean) value (4) and (ii) actual worm-burden (antigen) prevalence.

9 $$ {p}_W(t)=1-{h}_0(t) $$

In this analysis, a series of periodic MDA sessions was run until the 2 % target was reached or until an arbitrary time limit (set at 30 years) expired. At each MDA control session, a fraction of children (*f*_*c*_), and adults (*f*_*a*_) was scheduled for treatment to simulate non-participation in MDA delivery. MDA sessions were repeated at regular time intervals *τ* [years]. Drug efficacy was fixed at 75 % worm reduction, the rate we found most consistent with the available published data [38].

Simulated MDA outputs of interest to us included (i) prevalence reduction for communities (or specific groups) over time or within five years from the present (the year 2020) and (ii) for long term programs, the duration 0 < *T* ≤ 30 years required to achieve a target reduction to ≤ 2 % prevalence. In particular, we focused how *T* depends on the inter-treatment period *τ* and coverage fractions, {*T* = *T*(*τ*, *f*_*c*_, *f*_*a*_)}, along with which combinations (*τ*, *f*_*c*_, *f*_*a*_) would allow the program to reach its chosen target.

In most simulations reported, control inputs were allowed to vary over the following ranges: 0.5 < *τ* < 3; 0.5 ≤ *f*_*c*_ < 1; 0 ≤ *f*_*a*_ ≤ *f*_*c*_ . Special cases include community-wide treatment (CWT, where *f*_*a*_ = *f*_*c*_ ) and school-based treatment (SBT, where *f*_*a*_ = 0 < *f*_*c*_).

Two types of uncertainty enter our analysis and predictions: (i) uncertainty about in-host parasite biology, where each parameter choice carries an associated “likelihood weight”, (ii) variability in simulated egg-tests, whose outputs depend on random population sampling and irregular (NB-distributed) egg-release by hosts.

Uncertainty in predictions is managed as follows: In our simulations for each chosen MDA control, a treatment history is repeated multiple times for different choices of likely biological / transmission parameters and egg-test diagnostics. The resulting ranges of reported outcomes (*e.g.,* prevalence levels *p*_*E*_, *p*_*W*_, or required program duration, *T* ) are distributed quantities reflecting the underlying data/model input uncertainties. This allows statistical predictions of the estimated mean prevalence reductions and/or control duration, and the probability of attaining a particular target prevalence.

### 6. Short-term SCORE Project predictions and 2020 control

**Fig. 6:**
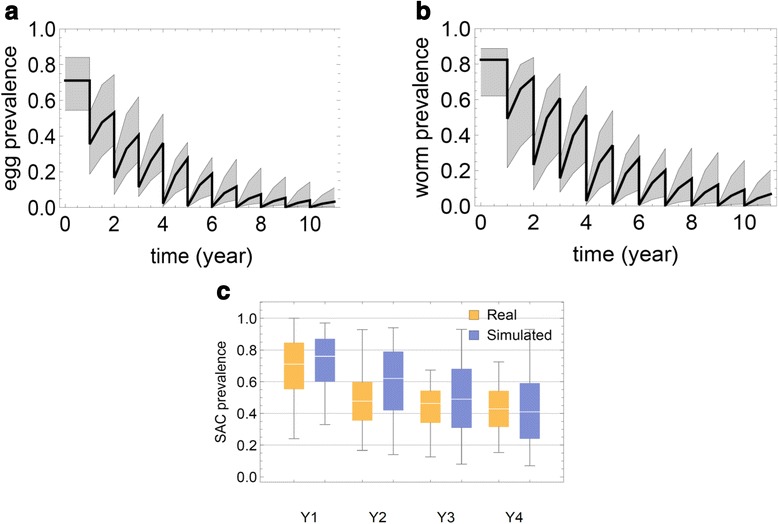
Egg prevalence projected in a 10 year MDA treatment simulation. Panel **a** shows the ensemble of estimated egg count-based prevalence values for 20 virtual communities throughout a 10 year treatment period of annual community-wide MDA. The median prevalence estimate is shown by the thick line, and the 25–75 % quantiles are indicated by the gray envelope; Panel **b** shows the corresponding estimates of worm-based prevalence, adjusting for the insensitivity and random components of egg counting; Panel **c** purple bars show the likely range of prevalence values for 9–12 year olds in 25 simulated villages in surveys performed before each of 4 yearly treatments (purple) in a SCORE-like program. Comparison to actual observed data from the SCORE Mozambique project is shown in yellow

Using characteristics and parameters derived for ‘virtual’ but typical high-risk and low-risk villages (model parameters calibrated based on our Kenya data), we ran simulation of SCORE Project *S. haematobium* control outcomes over a projected 10-year period (2010-2020) for a Mozambique-like environment, including demographic makeup and participation levels. We specifically focused on the subset of the trial using community-wide treatments (Fig. 6). For our initial simulations, we generated an ensemble of 20 virtual Mozambique-like communities and a hypothetical snail site with baseline (equilibrium) prevalences of susceptible, prepatent, and patent snails {x*, y*, z*} = {0.63, 0.35, 0.02} (taken from [23, 25], and Kariuki et al., unpublished data). Each community was divided into three age-groups (children, SAC, adults), each specified in terms of i) basal levels of egg-prevalence *p*_*E*_, ii) parasite biological parameters (*ρ*_0_, *k*_0_, *r*), and iii) population sizes *N* (used for random treatment/test sampling). The decision to use these specific age-range categories was based on the availability of in-depth individual-level data on infection and egg counts for the Kenya and Mozambique areas included in our analysis. In future, the SWB can be readily calibrated for other sites depending on available data, however, the accuracy and precision of predictions will depend on the depth of data support for any specific age group. Because adult participation in community surveys is often more sparse than that of SAC, we do not have as precise estimates of the range of egg outputs for the older members of most ‘typical’ *S. haematobium* affected communities. The work of Wilson, et al., [33] suggests that eggs/per worm decline in adulthood due to acquired anti-fecundity immunity, so that persistent infection with intermittent passage of eggs remains an important factor in continued transmission. Given these findings, we felt that using a 13+ aggregated age group was an appropriate compromise to reflect the reduced participation of older individuals and the post-12 yo shift from high intensity towards lower intensity infections. In the projected simulations, the SAC population numbers (*N*_*S*_) varied in the range 500–1000, while non-SAC populations were estimated from available census data (US Census Bureau International Database, at http://www.census.gov/population/international/data/idb/informationGateway.php) as *N*_*C*_ ≈ 2.8 × *N*_*S*_ (children) and *N*_*A*_ ≈ 12 × *N*_*S*_ (adults).

Expected prevalence levels for each village and demographic group were taken from SCORE data for sentinel 9–12 year old SAC, while the non-SAC groups were assigned random values of *p*_*E*_ in the range (0.1–0.4). Biological parameters (*ρ*_0_, *k*_0_, *r*) for each demographic group were chosen from the previously calibrated Kenyan posterior ensembles.

The coverage fractions in our simulations followed the typical SCORE coverage levels obtained over the first 4 years of the program, henceforth denoted Y1-Y4. In simulating the follow up period (through 2020), coverage levels were set to the level year Y4 of SCORE participation, *i.e.*, *f*_*SAC*_ = 0.8 , and 0.1 < *f*_*O*_ < 0.4 for other groups.

Although coverage rates {*f*} for each group are prescribed in our dynamic simulation, the MDA outcome could vary due to random selection of hosts in the SWB strata for treatment. The effect of random selection, however, diminished at high coverage rates *f* ≈ 1. To estimate egg-prevalence *p*_*E*_ at each control step, we used its expected value as calculated by Eq. (4).

## Results and discussion

To project the expected 2020 impact of MDA in a SCORE-like program setting, we ran an ensemble of 1000 realizations (50 random realization of each virtual village) and recorded their SAC prevalence as a function of time. Figure 6 shows simulated prevalence ensembles for 20 communities (median and quartiles) over a 10-year control period. In panel (a) we plotted the range of estimated mean egg-test prevalence outcomes from Eq. (4); in panel (b), we plotted estimated worm burden prevalence (based on antigen diagnostics) based on Eq. (3). As expected, prevalence of worm infection was slightly greater than egg count prevalence because if the insensitivity of egg count testing for low intensity infections. Prevalence was seen to decline after each annual round of MDA, but then to partially rebound due to reinfection. A period of 8 or more years of annual treatment was required to drop egg prevalence below 5 %. For validation, Fig. 6, panel (c) compares observed SCORE data for the 25 Mozambique CWT study communities with our simulated ensemble projections for CWT control over the initial 4-year period. Our model predictions showed a slower drop in *S. haematobium* prevalence during the initial phase (Y1-Y3), which caught up with the observed data in Y4. Not shown, in the projected follow-up period (Y5-Y10), we predict a further, significant reduction of prevalence (to ≈ 6 % by 2020) due to the relatively high treatment coverage levels achieved by Y4 (*f*_*SAC*_ = 0.8, 0.1 < *f*_*C*_; *f*_*A*_ < 0.4).

Of note, the model is currently parameterized for *S. haematobium* control using data from Kenya and Mozambique. It is possible that calibration and projected outputs will differ for *S. mansoni,* based on differences in snail host species and their related biological characteristics, and on different likelihood of transmission because of differences in egg excretion into water bodies (via feces vs. urine). We are currently examining this question in model parameterization based on SCORE data from *S. mansoni* communities in Kenya and Tanzania. A companion modeling paper in this issue (Anderson et al., What is required in terms of mass drug administration to interrupt the transmission of schistosome parasites in regions of endemic infection?) has examined impact of MDA on *S. mansoni* prevalence based on data from long baseline control studies in Kenya [39]. We expect that future work will allow for comparisons of the projections of both groups’ models, both for *S. haematobium* and *S. mansoni*.

### Long term target reduction

Our next goal was to explore the effect of a long-term MDA program targeted at reducing the prevalence to below 2 %. In particular, we were interested in the influence of the program’s chosen coverage fraction (*f*) and inter-treatment period (*τ*), and we looked for starting and operational conditions needed to reach the ≤ 2 % prevalence target within a 30-year time span. Specifically, we estimated the required program duration, *T,* as function of control inputs *T*(*f*, *τ*).

For these simulations, we generated an ensemble of 20 virtual communities of a Kenyan high transmission type area. Here, each simulated virtual community was made of 2 age-groups (children – 0-20 year-old, adults –20+), and their biological parameters (*ρ*_0_, *k*_0_, *r*) were chosen randomly from the calibration’s posterior ensembles. A hypothetical snail environment was used for coupled human-snail system, as in the shorter-term analysis described above. MDA coverage values for children were allowed to vary in the range 0.5 ≤ *f*_*C*_ ≤ 1 while adult coverage was kept at 75 % of children’s participation (*f*_*A*_ = 0.75*f*_*C*_) with the inter-treatment interval examined over a span of 0.5 < *τ* < 3 years.

Two types of outcomes for the sentinel child group were used in these long term simulations, egg prevalence (4) and worm prevalence (3). In either case, we assumed 20 % of children (out of total population 500) were screened for prevalence estimates before each MDA control step. When the ≤ 2 % children’s prevalence target was reached within 30-year period, we recorded time *T* and terminated the program. Otherwise it was left to run until the terminal value of 30 years, which was then assigned to the value of *T* for that simulation.

**Fig. 7:**
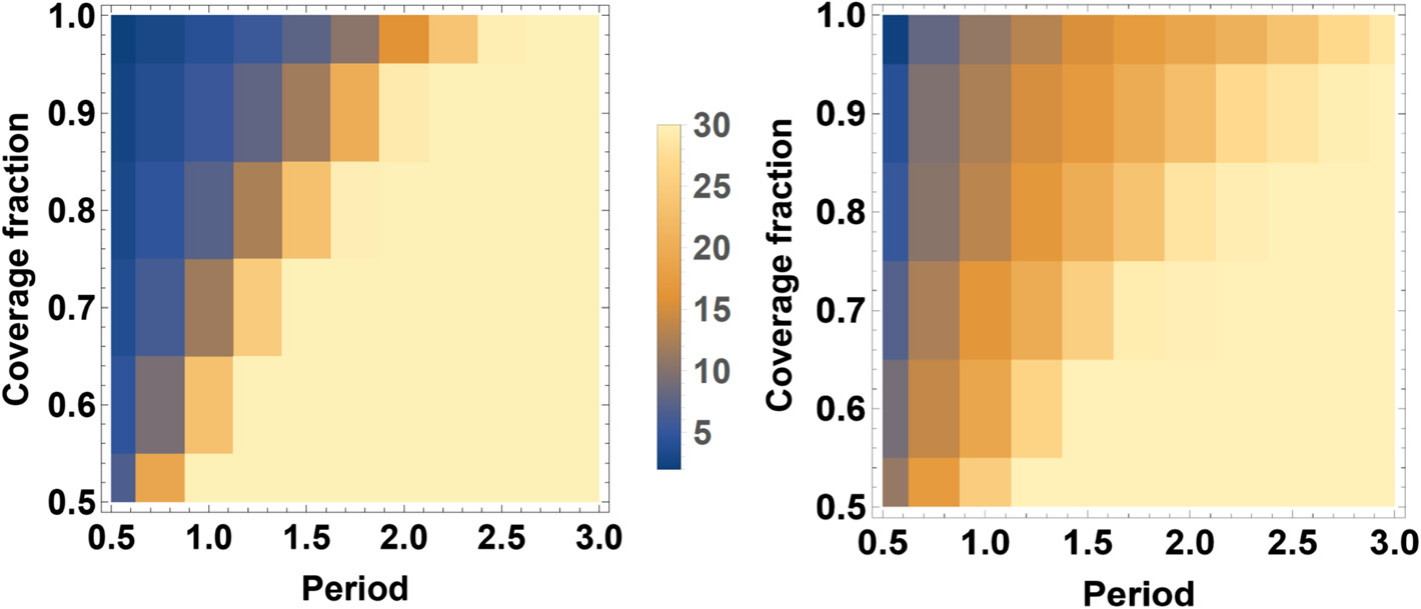
Heat map of the long term program duration (mean *T* ) required to reach ≤ 2 % *Schistosoma* prevalence. Two control strategies (CWT and SBT) are compared for typical Kenyan villages. Panel (**a**) show *T*(*f*_*C*_, *τ*) map for a high-risk (80 % baseline prevalence) treated with CWT; (**b**) lower risk village (30 % baseline prevalence) treated with SBT. The color scale in the center indicates the number of years needed to reach local *Schistosoma* infection prevalence of ≤ 2 %, as determined by egg-count diagnostics. The darkest color indicates the target will be reached in 5 years or fewer, the lightest color indicates the target is not reached in 30+ years of intervention. In both cases, long term simulations take into account predicted population growth for Kenya [34]

Each treatment regimen (*f*_*C*_, *f*_*A*_, *τ*) was repeated 50 times for each of the 20 virtual villages. The resulting 50 x 20 ensemble of observed values *T* (means and standard deviations) were recorded for each choice (*f*_*c*_, *τ*). Table 4 compiles T-values for egg-test diagnostics, while Table 5 does the same for worm-burden diagnostics. Figure 7 shows the results (mean values of *T* ) as a color map. Not surprisingly, based on the relative insensitivity of egg counting for low level infections, the results show that it takes longer to achieve truly low worm prevalence levels (Table 5) as compared to egg-count based prevalence levels (Table 4). Also, it shows that without substantially large coverage fraction (*f*) as well as a treatment frequency (1/ *τ*) of one year or less, prevalence cannot be reduced as low as 2 % within 30 years.

**Table 4:** Time to 2 % target prevalence based on egg-test diagnostics. Shown values represent ensemble mean ± SD of program duration (in years) required to reach a target *Schistosoma haematobium* infection prevalence ≤ 2 % (*T*(*f*_*C*_, *τ*)), for different choices of coverage (*f*_*c*_, columns) and inter-treatment periods (*τ*, rows). Upper panel A shows results for a high risk Kenyan village treated by CWT; Lower panel B – a low risk Kenyan village treated by SBT. Full results for different delivery strategies in high- and low-risk areas are shown in Additional file 5

A.	Community-wide MDA Coverage- High-risk village					
Interval	50 %	60 %	70 %	80 %	90 %	100 %
0.5	6.5 ± 1	4.5 ± 0.5	3.5 ± 0.5	3 ± 0.5	2.5 ± 0.5	2 ± 0.5
0.75	19 ± 8	9.5 ± 4	6 ± 1	4.5 ± 0.5	3.5 ± 0.5	3 ± 0.5
1	> 30	23.5 ± 8	11.5 ± 5.5	7 ± 1.5	5.5 ± 1	4 ± 0.5
1.25	> 30	> 30	25 ± 7.5	12.5 ± 5.5	7.5 ± 2	5.5 ± 1
1.5	> 30	> 30	> 30	23.5 ± 7.5	12 ± 6	7.5 ± 1.5
1.75	> 30	> 30	> 30	30 ± 2	20 ± 8	10.5 ± 5
2	> 30	> 30	> 30	> 30	29 ± 3	16 ± 8
2.25	> 30	> 30	> 30	> 30	> 30	24 ± 7.5
2.5	> 30	> 30	> 30	> 30	> 30	29.5 ± 2
2.75	> 30	> 30	> 30	> 30	> 30	> 30
3	> 30	> 30	> 30	> 30	> 30	> 30
ᅟ	ᅟ	ᅟ	ᅟ	ᅟ	ᅟ	ᅟ
B.	School-based MDA Coverage- Low-risk village					
Interval	50 %	60 %	70 %	80 %	90 %	100 %
0.5	12.5 ± 11.5	10.5 ± 11.5	8.5 ± 10.5	6.5 ± 10	5.5 ± 9.5	3.5 ± 6.5
0.75	18 ± 11	15 ± 11.5	13.5 ± 12.5	11.5 ± 12	11 ± 12.5	9.5 ± 12
1	25.5 ± 8	19.5 ± 10.5	17 ± 11.5	14.5 ± 12	13.5 ± 12.5	12 ± 12.5
1.25	> 30	26.5 ± 7.5	20.5 ± 10.5	17.5 ± 12	16 ± 12.5	14 ± 12.5
1.5	> 30	> 30	26 ± 7.5	20.5 ± 10.5	18 ± 11.5	16.5 ± 12.5
1.75	> 30	> 30	29.5 ± 3	24 ± 9	20.5 ± 11	18 ± 12
2	> 30	> 30	30 ± 2	28.5 ± 5	23 ± 9.5	19.5 ± 11.5
2.25	> 30	> 30	> 30	29.5 ± 2.5	27 ± 7	21 ± 10.5
2.5	> 30	> 30	> 30	> 30	28.5 ± 5	24 ± 9.5
2.75	> 30	> 30	> 30	> 30	29.5 ± 2.5	27 ± 6.5
3	> 30	> 30	> 30	> 30	> 30	29 ± 4.5

**Table 5:** Time to 2 % target prevalence based on antigen-detection diagnostics. Mean ± SD of program duration (in years) required to reach a target *Schistosoma haematobium* infection prevalence ≤ 2 % (*T*(*f*_*C*_, *τ*)). The panels and intervention values are the same as in Table 4 but using worm antigen-test diagnostics to identify post-treatment infection prevalence

A.	Community-wide MDA Coverage- High-risk village					
Interval	50 %	60 %	70 %	80 %	90 %	100 %
0.5	12 ± 6.5	7.5 ± 5.5	4.5 ± 0.5	3.5 ± 0.5	3 ± 0.5	2.5 ± 0.5
0.75	> 30	22.5 ± 9	11 ± 7	7.5 ± 5.5	4.5 ± 1	3.5 ± 0.5
1	> 30	> 30	27.5 ± 6.5	15 ± 9	8.5 ± 5.5	5 ± 1
1.25	> 30	> 30	> 30	27.5 ± 6.5	18 ± 10.5	8.5 ± 5.5
1.5	> 30	> 30	> 30	> 30	27.5 ± 6.5	17 ± 10
1.75	> 30	> 30	> 30	> 30	> 30	24.5 ± 8.5
2	> 30	> 30	> 30	> 30	> 30	> 30
2.25	> 30	> 30	> 30	> 30	> 30	> 30
2.5	> 30	> 30	> 30	> 30	> 30	> 30
2.75	> 30	> 30	> 30	> 30	> 30	> 30
3	> 30	> 30	> 30	> 30	> 30	> 30
ᅟ	ᅟ	ᅟ	ᅟ	ᅟ	ᅟ	ᅟ
B.	School-based MDA Coverage- Low-risk village					
Interval	50 %	60 %	70 %	80 %	90 %	100 %
0.5	17.5 ± 13.	16 ± 13.5	14 ± 13.5	13 ± 13.5	11 ± 13.	10.5 ± 13.
0.75	24.5 ± 9.5	19.5 ± 12.	17.5 ± 13.	16 ± 13.	14 ± 13.5	13.5 ± 14.
1	> 30	26 ± 8.5	21 ± 11.5	19 ± 12.5	16.5 ± 13.	15.5 ± 13.5
1.25	> 30	> 30	26 ± 8.5	21.5 ± 11.	19 ± 12.5	17.5 ± 13.
1.5	> 30	> 30	29 ± 4.	26 ± 8.5	21 ± 11.	19 ± 12.5
1.75	> 30	> 30	> 30	29 ± 4.5	25.5 ± 9.5	20 ± 11.5
2	> 30	> 30	> 30	> 30	28 ± 6.	23.5 ± 10.
2.25	> 30	> 30	> 30	> 30	> 30	25.5 ± 9.
2.5	> 30	> 30	> 30	> 30	> 30	28 ± 6.5
2.75	> 30	> 30	> 30	> 30	> 30	> 30
3	> 30	> 30	> 30	> 30	> 30	> 30

The next question asked was: What happens after the target is reached and control program stops? In past years, theoretical answers to this question have often been couched in terms of a putative “transmission breakpoint” infection levels [40–42]. Transmission breakpoints, when they exist, could prevent relaxation to pre-control levels of infection. Once breakpoint is reached (*e.g.*, via long term, intensive MDA) the system would relax to its lower and now stable “infection-free” state. Breakpoints are commonly predicted in MacDonald-type (MWB) model systems that include parasite mating (see [13, 15]), but from analysis of our calibrated SWB models, we believe them to occur only under exceptional circumstances [12]. In the absence of breakpoints the system is predicted to relax inevitably to its pre-control (baseline) equilibrium state. In fact, long term population growth could drive the equilibrium state even higher.

**Fig. 8:**
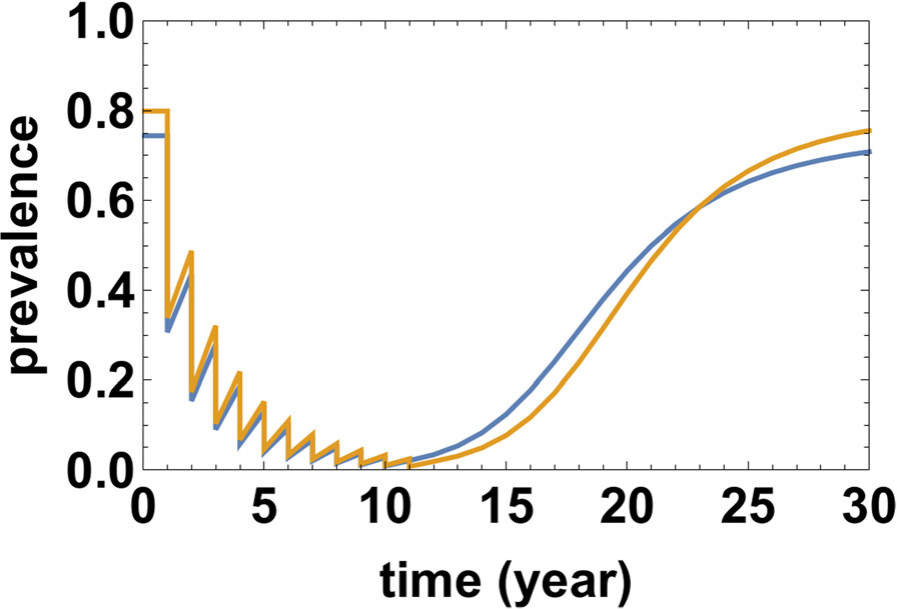
Typical MDA model histories given early target reduction (*T* = 6 − 8 years), but with subsequent relaxation to endemic equilibrium following suspension of program intervention. The two prevalence curves correspond to egg-test (darker blue line) and worm (antigen, lighter yellow line) diagnostics

Our simulations suggest that realistic environments like Kenya or Mozambique have no, or very low prevalence breakpoints (well below 2 %), perhaps too low to be of practical relevance. Figure 8 illustrates a typical MDA program that reaches the 2 %-target in relatively short time (6–8 years), but after termination prevalence ‘relaxes’ to its pre-control endemic levels by 22–24 years later.

## Conclusions

Our model simulations suggest the following conclusions about the currently advocated PCT programs: In a stable (stationary) transmission ecosystem, *Schistosoma* reproduction and transmission are sufficiently robust that the process of human infection continues, even under pressure from aggressive MDA. MDA alone is unlikely to interrupt transmission, and once mass treatment is suspended, the prevalence of human infection is likely to rebound to pre-control levels over a period of 25–30 years. MDA success in achieving very low levels of infection prevalence is highly dependent on treatment coverage and frequency within the local human population, and requires that both adults and school age children be included in drug delivery coverage.

The 2020 goals of the London Declaration and the WHO Roadmap are commendable, in that achieving 75 % coverage of at-risk school age children will significantly reduce the prevalence of *Schistosoma* infections, and hence reduce the risk of infection-associated morbidity. However, it is unlikely that the further programmatic objective of local or regional “elimination (where possible)” can be met in most locations without additional interventions *beyond* the basic school-based MDA now practiced in most endemic areas. Interval reassessments of persistent transmission, based on accurate and sensitive monitoring systems, will be needed to point out locations or regions where supplemental snail control and significant improvements in sanitation will be required to achieve the ultimate control of schistosomiasis by elimination of *Schistosoma* transmission.

## Additional files

Additional file 1:
**Further details of the SWB system.** (PDF 167 kb) Additional file 2:
**Random egg-release and simulated egg-test diagnostics.** (PDF 112 kb) Additional file 3:
**Likelihood estimates for simulation parameters, including egg test uncertainty.** (DOCX 69 kb) Additional file 4:
**Snail population and transmission dynamics.** (PDF 117 kb) Additional file 5:
**Complete heat maps and tables.** Long-term program duration (mean T) required to reach ≤ 2 % *Schistosoma haematobium* prevalence in simulated high- and lower-prevalence communities, based on calibration with data from Kenya (see text). Maps indicate the range of years required to reach the program target based on fractional coverage of the target population (Coverage, y-axis), and the interval between treatments (Period, x-axis), whether by community-wide treatment (CWT) or school-based treatment (SBT). Two sets of maps are provided—the first four are based on egg-count diagnostics, and the second four and are based on worm antigen detection. (PDF 1692 kb)

## Abbreviations

CWT: Community-wide treatment
FOI: Force of infection
MDA: Mass drug administration
MWB: Mean worm burden model
NB: Negative binomial distribution
NTD: Neglected tropical diseases
PCT: Preventive chemotherapy
SAC: School age children
SBT: School-based treatment
SCORE: Schistosomiasis consortium for operational research and evaluation
SWB: Stratified worm burden model
Y: Year
