# Supplementary material for: Modelling control of Schistosoma haematobium infection: predictions of the long-term impact of mass drug administration in Africa
Source: Parasit Vectors. 2015 Oct 22;8:529. doi: 10.1186/s13071-015-1144-3 (PMC4618728; doi:10.1186/s13071-015-1144-3)
Supplement: Additional file 1: — Further details of the SWB system. (PDF 167 kb) [file 13071_2015_1144_MOESM1_ESM.pdf]

Appendices

A. Further details of the SWB system

Host population $H=\sum_{k=0}^{n} h_k$ is divided into burden strata $\{h_k\}$ by their worm load (*w* = # adult worms): $k\,\Delta w \le w < (k+1)\Delta w$ for h_k. The partition is determined by worm-step $\Delta w \ge 1$, that derives from a hypothetical *mating threshold*. So h_0 are infection-free (no mated couples), while for h_k ($k \ge 1$), its mated count (expected number of couples) is given by equation (14) of Additional File 2, Appendix B. The transitions among strata

$$\begin{array}{ccccccc} \downarrow S_0 & & \downarrow S_1 & & & & \downarrow S_n \\ h_0(t) & \underset{\gamma_1}{\overset{\lambda}{\rightleftharpoons}} & h_1(t) & \underset{\gamma_2}{\overset{\lambda}{\rightleftharpoons}} \ldots & & \underset{\gamma_n}{\overset{\lambda}{\rightleftharpoons}} & h_n(t) \\ \downarrow \mu & & \downarrow \mu & & & & \downarrow \mu \end{array}$$

are determined by FOI λ (= rate of worm accumulation), resolution rates $\gamma_k = k\,\gamma$ (based on estimated natural worm mortality γ), and population turnover rate μ (human mortality, maturation, migration, etc.). Source terms S_k represent demographic inputs from related population groups, *e.g.*, for infants $S_0 = b_H$ (birth rate), while $S_{k\ge 1} = 0$ (representing the fact that all newborns are infection-free), while adult sources come from maturing childhood strata. Dynamic variables $\vec{h}(t)=\{h_k(t)\}$ obey a coupled differential system

$$\begin{cases} \dfrac{dh_0}{dt} = -(\lambda+\mu)h_0 + \gamma_1 h_1 + S_0 \\ \vdots \\ \dfrac{dh_i}{dt} = \lambda h_{i-1} - (\lambda+\gamma_i+\mu)h_i + \gamma_{i+1}h_{i+1} + S_i \\ \vdots \\ \dfrac{dh_n}{dt} = \lambda h_{n-1} - (\gamma_n+\mu)h_n + S_n \end{cases} \qquad (10)$$

We write it using vector notation as

$$\frac{d\vec{h}}{dt} = A(\lambda,\mu,\gamma)\cdot\vec{h} + \vec{S} \quad , \tag{11}$$

with FOI (transition) matrix

$$A(\lambda,\mu,\gamma) = \begin{bmatrix} -(\lambda+\mu) & \gamma_1 & & & & \\ \lambda & \ddots & \ddots & & & \\ & \lambda & -(\lambda+\gamma_i+\mu) & \gamma_{i+1} & & \\ & & \ddots & \ddots & \gamma_n & \\ & & & \lambda & -(\gamma_n+\mu) & \end{bmatrix}.$$

Reduced systems, like the MacDonald mean worm burden (MWB) models can be derived as moment equations of system (10) (see [12]).

For age-structured populations (child + adult), each subgroup has its own SWB, with specific FOI - λ^d, turnover μ^d, and source $\overrightarrow{S^d}$. The children's' source $\overrightarrow{S^C} = \{b_C, 0, 0, ...\}$ comes from newborns (b_C = per capita birth rate) entering the uninfected h_0 -stratum. The adult source is contributed by aging children,

$$\overrightarrow{S^A} = \{S_k^A\} = \frac{\tau}{\gamma}\frac{H_C}{H_A}\overrightarrow{h^C} \tag{12}$$

Here $\{H_C, H_A\}$ are populations of two groups, τ - maturation rate (= 1/maturation age). Child and adult SWB variables $\overrightarrow{h^C}$, $\overrightarrow{h^A}$ are coupled via

$$\begin{cases} \frac{d}{dt}\overrightarrow{h^C} = A(\lambda^C, \mu^C, \gamma)\cdot\overrightarrow{h^C} + \overrightarrow{S^C} \\ \frac{d}{dt}\overrightarrow{h^A} = A(\lambda^A, \mu^A, \gamma)\cdot\overrightarrow{h^A} + \overrightarrow{S^A} \end{cases} \tag{13}$$
