# Supplementary material for: Modelling control of Schistosoma haematobium infection: predictions of the long-term impact of mass drug administration in Africa
Source: Parasit Vectors. 2015 Oct 22;8:529. doi: 10.1186/s13071-015-1144-3 (PMC4618728; doi:10.1186/s13071-015-1144-3)
Supplement: Additional file 2: — Random egg-release and simulated egg-test diagnostics. (PDF 112 kb) [file 13071_2015_1144_MOESM2_ESM.pdf]

B. Random egg-release and simulated egg-test diagnostics

Two factors determine egg-release by human hosts: the number of fertilized females ϕ_k (in k-th stratum), and worm fecundity ρ_k. For estimating mated worm counts we follow [16, 12] which gives

$$\phi(w) = \frac{w}{2}\left[1 - 2^{-w}\binom{w}{w/2}\right]; \text{ or } \ \phi_k = \phi(k\,\Delta w) \qquad (14)$$

for h_k -stratum. For worm fecundity, $\rho(w)$ or ρ_k we assume exponential fall-off ("crowding effect"), given by function (1)

$$\rho(w) = \rho_0\, e^{-w/w_0}; \text{ or } \ \rho_k = \rho(k\,\Delta w) = \rho_0\, e^{-k/k_0} \qquad (15)$$

with maximal value ρ_0, and threshold $k_0 = \frac{w_0}{\Delta w}$. Equations (14) - (15) predict mean egg-release by individual host in h_k

$$E_k = \rho_k\, \phi_k \qquad (16)$$

Observed egg-counts in diagnostic test data are typically overdispersed, while repeated tests on individual hosts show high day-to-day variability. So following [27], we choose to describe the egg-release process as random negative binomial (NB) variable in our current SWB approach. Specifically, a mated female in h_k -stratum is assumed to release random NB-count with *mean* ρ_k and aggregation parameter *r* (estimated based on our calibration). The resulting egg-release by h_k -hosts (carrying ϕ_k mated worms) is also NB with mean $E_k = \rho_k\, \phi_k$, and aggregation $r_k = r\, \phi_k$. For the combined SWB

community $\{h_k\}$, observed egg-test results are random samples drawn from a mixed NB distribution $D_M = \sum_{k=0}^{n} h_k \, NB\left(E_k \mid r \, \phi_k\right)$, as indicated in Figure 1.
