# Supplementary material for: Modelling control of Schistosoma haematobium infection: predictions of the long-term impact of mass drug administration in Africa
Source: Parasit Vectors. 2015 Oct 22;8:529. doi: 10.1186/s13071-015-1144-3 (PMC4618728; doi:10.1186/s13071-015-1144-3)
Supplement: Additional file 3: — Likelihood estimates for simulation parameters, including egg test uncertainty. (DOCX 69 kb) [file 13071_2015_1144_MOESM3_ESM.docx]

# Appendix C: Likelihood estimates for simulation parameters, including egg test uncertainty

The goal of the calibration scheme was to assign a likelihood weight to each suite of parameter choices for the model system. The suite was made up of combined biological and transmission parameters of the model. The calculated likelihood weights for each tested suite were based on an estimated distance function between the model’s simulated test results (utilizing the chosen suite of parameters) and observed data from field studies.

Both data sets, real and simulated, are represented by their discretized (binned) distribution sequences $E_{S}=\left\{e_{0};e_{1};...;e_{20}\right\}$(for the simulated data), and $E_{D}=\left\{d_{0};d_{1};...\right\}$(for the observed data). Simulation of diagnostic test outcomes, as monitored in control programs, involved two random steps: i) hosts from SWB strata were randomly drawn with probabilities $\left\{h_{n}\right\}$, ii) an egg count was estimated for each host following an hypothesized random-negative binomial (NB) egg release profile. We think of $E_{D}$ as a random realization of such a process, so to compare a simulated $E_{S}$ with an observed $E_{D}$, we generated for each parameter suite chosen ($\lambda,p_{B}$), an ensemble $E\text{=}\left\{E_{S}\right\}$ (several hundred simulated community egg-tests), and estimated the departure of ensemble mean $\bar{E}$ from $E_{D}$ relative to ensemble covariance $\sigma$.

The truncated covariance matrix, $\sigma$, of test ensemble $E$ is represented by the first few principle components, corresponding to its largest eigenvalues: $\lambda_{1}>\lambda_{2}>...>\lambda_{m}$ , truncated at $\lambda_{m}\geq.001 \lambda_{1}$(typically 3-5 principal components). The corresponding “likelihood distance” between $E$ and $E_{D}$ is defined as

$$d\left(E,E_{D}\right)=\left(\bar{E}-E_{D}\right)\cdot\sigma^{-1}\cdot\left(\bar{E}-E_{D}\right)$$

with quasi-inverse matrix $\sigma^{-1}$. In Bayesian terminology, the likelihood estimate can be viewed as the conditional probability$P\left(E_{D}|E\right)$. For normal (Gaussian) distributions, likelihood estimates depend on a suitable square-mean distance function. Assuming $E_{S}$ is “approximately” normal, we estimate the distance (error) function$d\left(E_{S},E_{D}\right)$ , using principal component analysis of the covariance matrix $\sigma^{-1}$of $E_{S}$, as above.

Distance gives rise to likelihood weights for any parameter choices $\vec{p}=\left(\lambda,p_{B}\right)$

$$W\left(\lambda,p_{B}\right)=\exp\left(-\frac{d\left(E\left(\lambda,p_{B}\right),E_{D}\right)}{20}\right)$$

Likelihood weights $\left\{W\left(\lambda,p_{B}\right)\right\}$ define a posterior empirical distribution for $\left(\lambda,p_{B}\right)$ -space reflecting the central tendency and uncertainty of model predictions.
