# Supplementary material for: Modelling control of Schistosoma haematobium infection: predictions of the long-term impact of mass drug administration in Africa
Source: Parasit Vectors. 2015 Oct 22;8:529. doi: 10.1186/s13071-015-1144-3 (PMC4618728; doi:10.1186/s13071-015-1144-3)
Supplement: Additional file 4: — Snail population and transmission dynamics. (PDF 117 kb) [file 13071_2015_1144_MOESM4_ESM.pdf]

C. Snail population and transmission dynamics

Dynamic variables for the snail component of our model are population densities (per unit habitat) of *x*- susceptible snails; *y*- prepatent infected snails; and *z*-patent infected snails that are shedding *Schistosoma* cercariae; whereby *N*=*x*+*y*+*z*- total snails. All variables in this scheme represent adult populations, and the relation among them is shown in following SEI diagram

$$\begin{array}{ccccccc} \beta & & \Lambda & & cr & \\ \rightarrow & x & \rightleftarrows & y & \rightarrow & z \\ & \downarrow & (1-c)r & \downarrow & & \downarrow \\ & \nu & & \nu & & \nu \end{array}$$

We assume logistic population growth $\beta = \beta_0 (x+y)(1-N/K)$, with maximal reproduction rate β_0 and carrying capacity *K*, and fixed snail mortality ν . Only susceptible and prepatent snails ($x+y$) can reproduce and contribute to growth. The transmission (SEI) part of the system has snail FOI, Λ , determined by the infective human population; prepatency rate r (period 1/r), and patency conversion fraction *c*. The combined population growth-SEI dynamics is described by 3 differential equations

$$\frac{dx}{dt} = \beta - \Lambda x - \nu x + r(1-c)y$$
$$\frac{dy}{dt} = \Lambda x - (r+\nu)y$$
$$\frac{dz}{dt} = c r y - \nu z$$

Parameter values and ranges for the snail system are given in Table 2. The force of snail infection, Λ , is a nonlinear (saturated) function of the total egg release by the human host population per snail

$$\Lambda = \Lambda_0 \left(1 - \exp\left[-b\,\omega \frac{H\,E}{N}\right]\right) \quad (17)$$

H – host population size, *E* – mean per capita egg-release by hosts

$$E = E\left(\rho_0, k_0\right) = \sum_k \rho_k \phi_k \, h_k \quad (18)$$

The key inputs in Λ are sporocyst establishment rate Λ_0, and transmission coefficient $B = \alpha\,\beta_M$ (product of miracidium coefficient, β_M , times snail susceptibility, α).

In coupled human-snail systems, human FOI $\lambda = a\,\omega\,z$, is the product of exposure (contact) rate ω, patent snail density z, and transmission coefficient A that depends on cercarial production/mortality, and the probability of worm establishment in the human host.

Two transmission rates $A = a\,\omega;\ B = b\,\omega$ of the coupled system could be estimated from the known demographic and infection data for a given environment.
