# Supplementary material for: Modelling control of Schistosoma haematobium infection: predictions of the long-term impact of mass drug administration in Africa
Source: Parasit Vectors. 2015 Oct 22;8:529. doi: 10.1186/s13071-015-1144-3 (PMC4618728; doi:10.1186/s13071-015-1144-3)
Supplement: Additional file 5: — Complete heat maps and tables. Long-term program duration (mean T) required to reach ≤ 2 % Schistosoma haematobium prevalence in simulated high- and lower-prevalence communities, based on calibration with data from Kenya (see text). Maps indicate the range of years required to reach the program target based on fractional coverage of the target population (Coverage, y-axis), and the interval between treatments (Period, x-axis), whether by community-wide treatment (CWT) or school-based treatment (SBT). Two sets of maps are provided—the first four are based on egg-count diagnostics, and the second four and are based on worm antigen detection. (PDF 1692 kb) [file 13071_2015_1144_MOESM5_ESM.pdf]

CWT / high risk village / egg-test

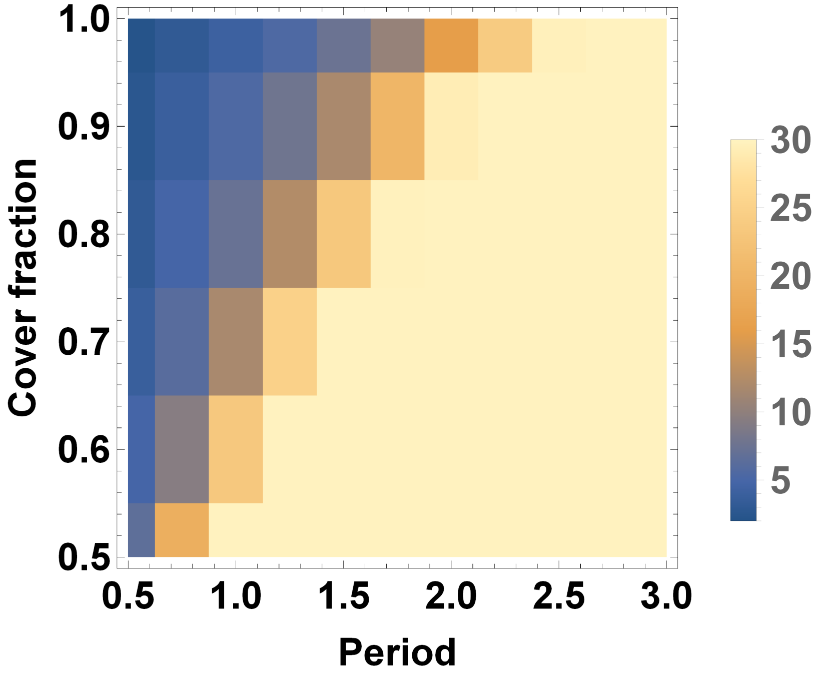

	0.5	0.6	0.7	0.8	0.9	1.
0.5	6.5±1.	4.5±0.5	3.5±0.5	3.±0.5	2.5±0.5	2.±0.5
0.75	19.±8.	9.5±4.	6.±1.	4.5±0.5	3.5±0.5	3.±0.5
1.	30.±0.	23.5±8.	11.5±5.5	7.±1.5	5.5±1.	4.±0.5
1.25	30.±0.	30.±0.	25.±7.5	12.5±5.5	7.5±2.	5.5±1.
1.5	30.±0.	30.±0.	30.±0.	23.5±7.5	12.±6.	7.5±1.5
1.75	30.±0.	30.±0.	30.±0.	30.±2.	20.±8.	10.5±5.
2.	30.±0.	30.±0.	30.±0.	30.±0.	29.±3.	16.±8.
2.25	30.±0.	30.±0.	30.±0.	30.±0.	30.±0.	24.±7.5
2.5	30.±0.	30.±0.	30.±0.	30.±0.	30.±0.	29.5±2.
2.75	30.±0.	30.±0.	30.±0.	30.±0.	30.±0.	30.±0.
3.	30.±0.	30.±0.	30.±0.	30.±0.	30.±0.	30.±0.

SBT / high risk village / egg-test

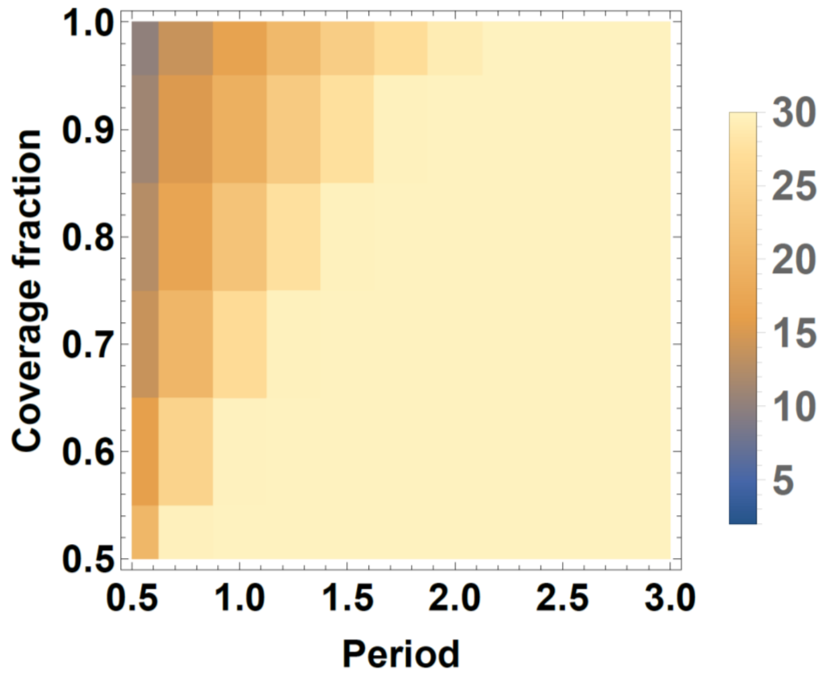

	0.5	0.6	0.7	0.8	0.9	1.
0.5	19.5±10.	15.5±10.5	13.±10.5	11.5±10.	10.±10.	8.5±9.5
0.75	30.±1.5	25.±8.	19.5±10.	16.5±10.5	14.5±10.5	12.5±10.5
1.	30.±0.	30.±1.	26.5±7.	22.±9.5	18.5±10.	16.±10.5
1.25	30.±0.	30.±0.	30.±1.	27.5±5.5	23.5±9.	20.±10.
1.5	30.±0.	30.±0.	30.±0.	30.±1.5	27.5±6.	24.±8.5
1.75	30.±0.	30.±0.	30.±0.	30.±0.	30.±1.5	27.±6.5
2.	30.±0.	30.±0.	30.±0.	30.±0.	30.±0.	29.±3.5
2.25	30.±0.	30.±0.	30.±0.	30.±0.	30.±0.	30.±0.
2.5	30.±0.	30.±0.	30.±0.	30.±0.	30.±0.	30.±0.
2.75	30.±0.	30.±0.	30.±0.	30.±0.	30.±0.	30.±0.
3.	30.±0.	30.±0.	30.±0.	30.±0.	30.±0.	30.±0.

CWT / low risk village / egg-test

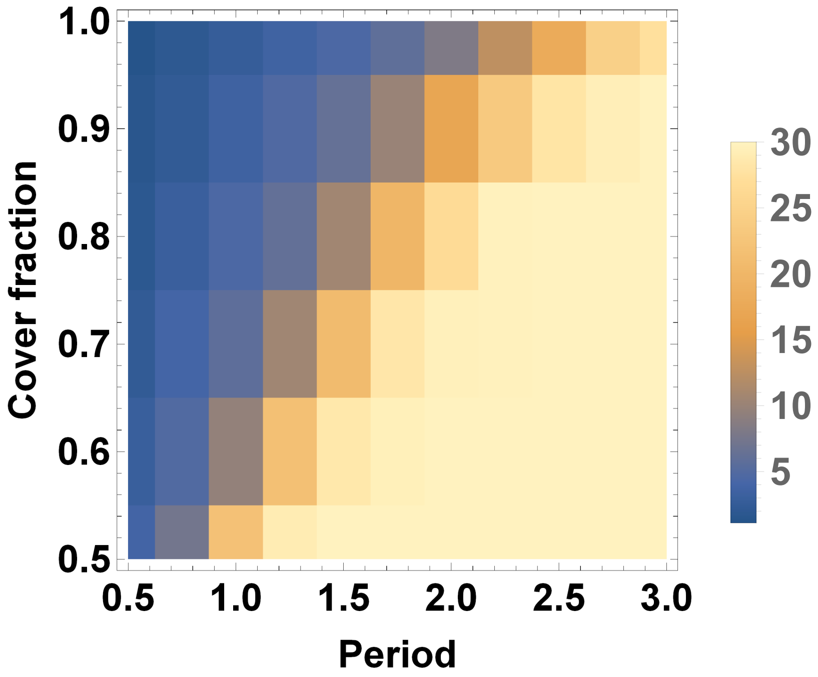

	0.5	0.6	0.7	0.8	0.9	1.
0.5	3.5±1.	3.±0.5	2.±0.5	1.5±0.5	1.5±0.5	1.±0.5
0.75	7.±3.5	5.±1.	4.±1.	3.±0.5	2.±0.5	2.±0.5
1.	22.±10.	9.5±5.5	5.5±1.5	4.5±1.	3.5±0.5	2.5±0.5
1.25	29.±4.	21.5±9.5	10.5±6.	6.±2.	4.5±1.5	3.5±1.
1.5	30.±0.	28.5±5.	21.±9.5	10.5±6.5	6.±2.	4.5±1.5
1.75	30.±0.	29.5±2.5	28.5±5.	20.±10.	10.±6.	6.±2.
2.	30.±0.	30.±0.	29.5±2.5	26.5±7.5	17.±10.	8.±5.5
2.25	30.±0.	30.±0.	30.±1.5	30.±2.	23.5±9.5	12.5±9.
2.5	30.±0.	30.±0.	30.±0.	30.±0.	28.±5.5	18.±10.
2.75	30.±0.	30.±0.	30.±0.	30.±0.	29.5±3.	24.5±8.5
3.	30.±0.	30.±0.	30.±0.	30.±0.	30.±0.	27.5±7.

SBT / low risk village / egg-test

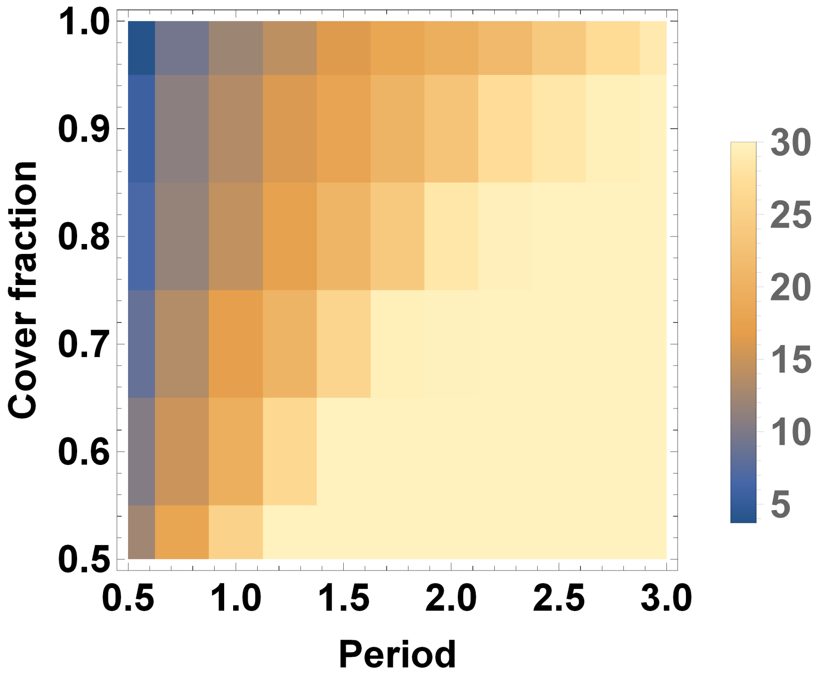

	0.5	0.6	0.7	0.8	0.9	1.
0.5	12.5±11.5	10.5±11.5	8.5±10.5	6.5±10.	5.5±9.5	3.5±6.5
0.75	18.±11.	15.±11.5	13.5±12.5	11.5±12.	11.±12.5	9.5±12.
1.	25.5±8.	19.5±10.5	17.±11.5	14.5±12.	13.5±12.5	12.±12.5
1.25	30.±0.	26.5±7.5	20.5±10.5	17.5±12.	16.±12.5	14.±12.5
1.5	30.±0.	30.±0.	26.±7.5	20.5±10.5	18.±11.5	16.5±12.5
1.75	30.±0.	30.±0.	29.5±3.	24.±9.	20.5±11.	18.±12.
2.	30.±0.	30.±0.	30.±2.	28.5±5.	23.±9.5	19.5±11.5
2.25	30.±0.	30.±0.	30.±0.	29.5±2.5	27.±7.	21.±10.5
2.5	30.±0.	30.±0.	30.±0.	30.±0.	28.5±5.	24.±9.5
2.75	30.±0.	30.±0.	30.±0.	30.±0.	29.5±2.5	27.±6.5
3.	30.±0.	30.±0.	30.±0.	30.±0.	30.±0.	29.±4.5

CWT / high risk village / antigen-test

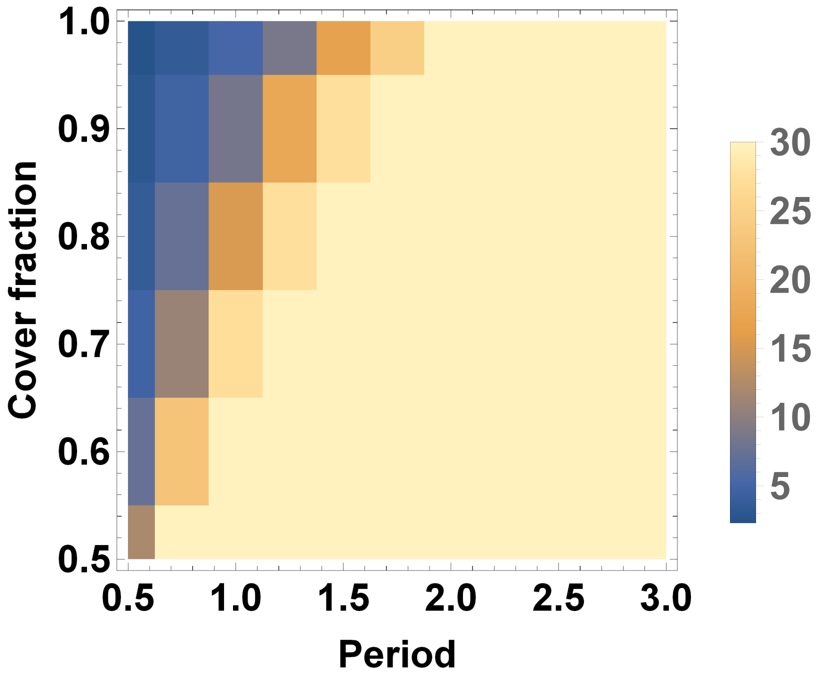

	0.5	0.6	0.7	0.8	0.9	1.
0.5	12.±6.5	7.5±5.5	4.5±0.5	3.5±0.5	3.±0.5	2.5±0.5
0.75	30.±0.	22.5±9.	11.±7.	7.5±5.5	4.5±1.	3.5±0.5
1.	30.±0.	30.±0.	27.5±6.5	15.±9.	8.5±5.5	5.±1.
1.25	30.±0.	30.±0.	30.±0.	27.5±6.5	18.±10.5	8.5±5.5
1.5	30.±0.	30.±0.	30.±0.	30.±0.	27.5±6.5	17.±10.
1.75	30.±0.	30.±0.	30.±0.	30.±0.	30.±0.	24.5±8.5
2.	30.±0.	30.±0.	30.±0.	30.±0.	30.±0.	30.±0.
2.25	30.±0.	30.±0.	30.±0.	30.±0.	30.±0.	30.±0.
2.5	30.±0.	30.±0.	30.±0.	30.±0.	30.±0.	30.±0.
2.75	30.±0.	30.±0.	30.±0.	30.±0.	30.±0.	30.±0.
3.	30.±0.	30.±0.	30.±0.	30.±0.	30.±0.	30.±0.

SBT / high risk village / antigen-test

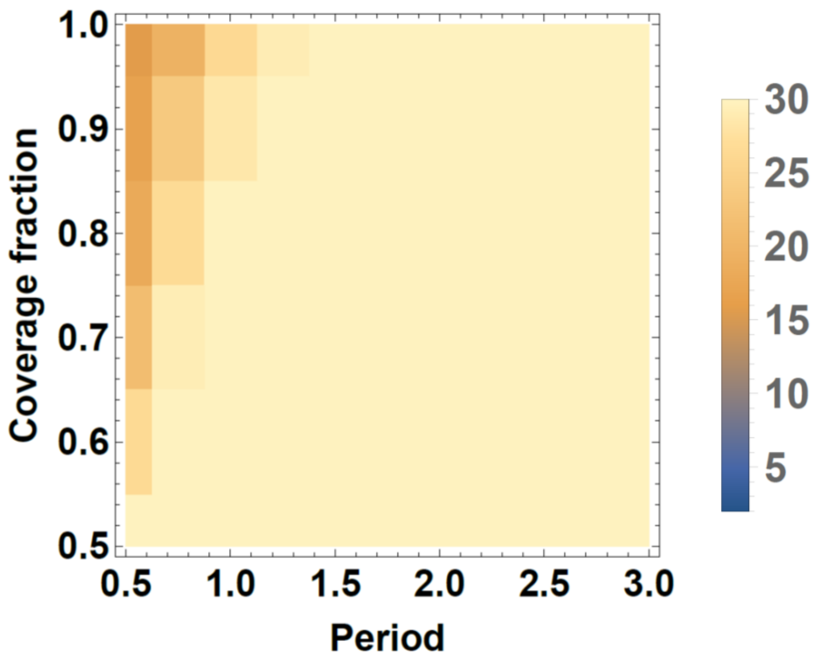

	0.5	0.6	0.7	0.8	0.9	1.
0.5	30.±0.	26.5±7.5	21.±9.5	17.5±10.	16.±11.	15.±11.5
0.75	30.±0.	30.±0.	29.±3.5	26.5±7.5	23.±9.5	19.±10.
1.	30.±0.	30.±0.	30.±0.	30.±0.	28.5±5.	26.±8.
1.25	30.±0.	30.±0.	30.±0.	30.±0.	30.±0.	29.±3.5
1.5	30.±0.	30.±0.	30.±0.	30.±0.	30.±0.	30.±0.
1.75	30.±0.	30.±0.	30.±0.	30.±0.	30.±0.	30.±0.
2.	30.±0.	30.±0.	30.±0.	30.±0.	30.±0.	30.±0.
2.25	30.±0.	30.±0.	30.±0.	30.±0.	30.±0.	30.±0.
2.5	30.±0.	30.±0.	30.±0.	30.±0.	30.±0.	30.±0.
2.75	30.±0.	30.±0.	30.±0.	30.±0.	30.±0.	30.±0.
3.	30.±0.	30.±0.	30.±0.	30.±0.	30.±0.	30.±0.

CWT / low risk village / antigen-test

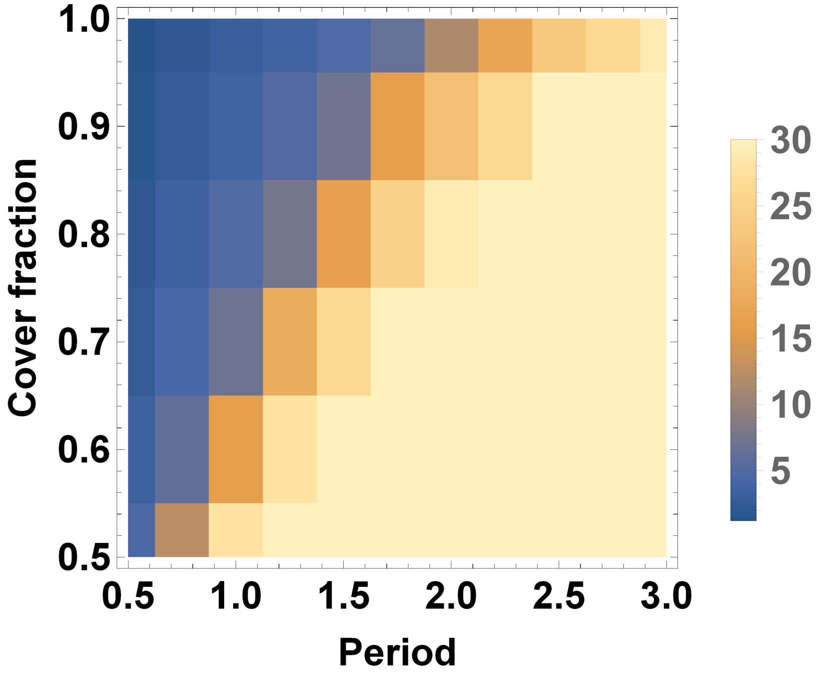

	0.5	0.6	0.7	0.8	0.9	1.
0.5	4.5±1.	3.±1.	2.5±0.5	2.±0.5	1.5±0.5	1.±0.5
0.75	12.5±9.	6.±1.5	4.±1.	3.±1.	2.5±0.5	2.±0.5
1.	28.±6.5	15.5±10.	7.±2.5	5.±1.5	3.5±1.	3.±0.5
1.25	30.±0.	28.±6.5	18.±11.	7.5±2.5	5.±1.5	3.5±1.
1.5	30.±0.	30.±0.	26.±8.	16.±10.5	7.±2.5	4.5±1.5
1.75	30.±0.	30.±0.	30.±0.	25.±9.	15.5±11.	6.5±2.5
2.	30.±0.	30.±0.	30.±0.	29.±4.	21.5±11.	11.5±8.5
2.25	30.±0.	30.±0.	30.±0.	30.±0.	26.±8.	17.±11.
2.5	30.±0.	30.±0.	30.±0.	30.±0.	30.±0.	23.5±10.5
2.75	30.±0.	30.±0.	30.±0.	30.±0.	30.±0.	26.±8.
3.	30.±0.	30.±0.	30.±0.	30.±0.	30.±0.	29.±4.5

SBT / low risk village / antigen-test

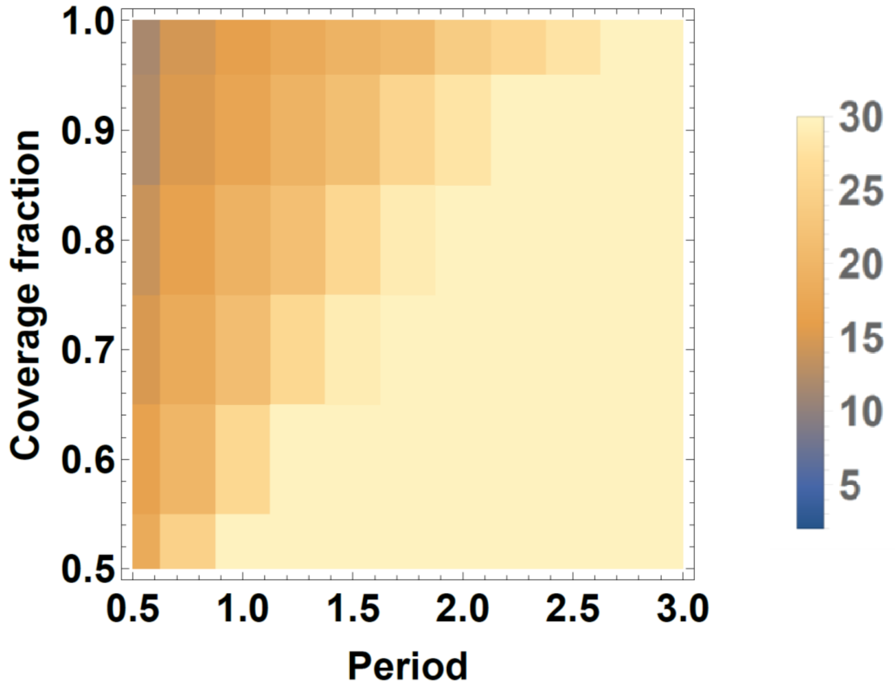

	0.5	0.6	0.7	0.8	0.9	1.
0.5	17.5±13.	16.±13.5	14.±13.5	13.±13.5	11.±13.	10.5±13.
0.75	24.5±9.5	19.5±12.	17.5±13.	16.±13.	14.±13.5	13.5±14.
1.	30.±0.	26.±8.5	21.±11.5	19.±12.5	16.5±13.	15.5±13.5
1.25	30.±0.	30.±0.	26.±8.5	21.5±11.	19.±12.5	17.5±13.
1.5	30.±0.	30.±0.	29.±4.	26.±8.5	21.±11.	19.±12.5
1.75	30.±0.	30.±0.	30.±0.	29.±4.5	25.5±9.5	20.±11.5
2.	30.±0.	30.±0.	30.±0.	30.±0.	28.±6.	23.5±10.
2.25	30.±0.	30.±0.	30.±0.	30.±0.	30.±0.	25.5±9.
2.5	30.±0.	30.±0.	30.±0.	30.±0.	30.±0.	28.±6.5
2.75	30.±0.	30.±0.	30.±0.	30.±0.	30.±0.	30.±0.
3.	30.±0.	30.±0.	30.±0.	30.±0.	30.±0.	30.±0.
